# Genome-Wide Identification and Functional Studies of the APX Gene Family in Oat (*Avena sativa* L.)

**DOI:** 10.3390/life16030494

**Published:** 2026-03-18

**Authors:** Conghui Li, Lijuan Zhao, Xinmei Li, Xinyu He, Yuhao Niu, Guangyin Wang, Lijun Cheng, Siyue He, Yi Zhang, Haitao Liu

**Affiliations:** 1 School of Biological Engineering, Huainan Normal University, Huainan 232038, China; 2 Key Laboratory of Bioresource and Environmental Biotechnology of Anhui Higher Education Institutes, Huainan Normal University, Huainan 232038, China

**Keywords:** oat (*Avena sativa*), ascorbate peroxidase, bioinformatics, light response

## Abstract

Ascorbate peroxidase (APX) plays a crucial role in both the removal of hydrogen peroxide and chloroplast development in response to light. To clarify the function of the APX gene family in oat (*Avena sativa* L.), we identified the family members and systematically analyzed their characteristics, phylogenetic relationships, promoter *cis*-elements, and expression patterns. Overall, 27 oat APX (AsAPX) members were identified in oat, and all encoded products had a peroxidase or peroxidase-like heptapeptide structure and motif. The genes were distributed unevenly across 15 chromosomes, with amino acid sequences ranging from 112 to 510 and molecular weights varying between 11.83 and 55.45 kDa. A phylogenetic analysis revealed that AsAPXs can be categorized into five branches, while an intra-group syntenic analysis identified 17 pairs of duplicate segments. Furthermore, 41 *cis*-element recognition sites were identified in the promoter regions of *AsAPX* genes, primarily comprising light-responsive and phytohormone-responsive elements. Moreover, qRT-PCR results indicated that *AsAPX* genes respond to light. Based on these results, our research establishes a foundation for exploration of *AsAPX* gene functionality and offers light-inducible candidate genes for chloroplast development to enhance *A. sativa* and improve crop production.

## 1. Introduction

Oat (*Avena sativa* L.), an annual cereal and fodder crop, belongs to the grass family. It is widely cultivated worldwide due to its high stress resistance and feed value, including in North America, Russia, Canada, and Europe, and China also contributes a growing share of total oat production [1,2]. Currently, the global production of oat ranks seventh among cereals, and global and Chinese oat production reached approximately 22.3 and 0.85 million metric tons, respectively, in 2024 (FAO, 2024, http://www.fao.org/faostat/en/, accessed on 14 February 2026). Oats have also gradually become an important source of roughage for cattle and sheep farming, under the backdrop of a continuously developing livestock industry.

Light, as the primary energy source for plants, plays a crucial role in regulating plant growth and development throughout their life cycle [3]. When the etiolated seedlings, which grow in darkness, emerge from the soil, they perform photomorphogenesis to facilitate photosynthesis and chloroplast development [3]. However, these processes are accompanied by the generation of reactive oxygen species (ROS). Excessive ROS damage cellular systems, ultimately leading to cell death and significantly inhibiting photosynthesis, which compromises crop yield and poses a risk to food security [4]. Consequently, how to increase crop photosynthesis and further improve grain production has emerged as a pressing scientific problem.

Ascorbate peroxidase (APX; EC, 1.11.1.11) is one of the main antioxidant enzymes involved in ROS scavenging mechanisms [5]. The APX family is divided into the enzymatically active canonical APX isoforms and the catalytically divergent ascorbate peroxidase-related protein (APX-R) and ascorbate peroxidase-like protein (APX-L) [5]. Canonical APXs are further divided into four classes according to the subcellular localization of their proteins in the cytoplasm, peroxisomes, chloroplasts, and mitochondria [5]. In contrast, APX-R and APX-L are evolutionarily derived clades, resulting in a loss of or drastic reduction in canonical ascorbate peroxidase activity due to a lack of critical ascorbate binding residues, which serve as a specific electron donor when compared to classical APX [6]. In higher plants, canonical APXs are crucial in both chloroplasts and mitochondria due to the continuous production of ROS during photosynthesis and respiration, respectively [7]. These genes have been identified and annotated in various plants [5]. In *Arabidopsis thaliana*, the APX family includes eight members: three cytosolic (*AtAPX1*, *-2*, and *-6*), three peroxisomal (*AtAPX3*, *-4*, and *-5*), and two chloroplastic (*AtsAPX* and *AttAPX*) members [8,9]. Rice (*Oryza sativa* L.) also contains eight APX genes, including two cytoplasmic isoforms (*OsAPX1* and *-2*), two peroxidase isoforms (*OsAPX3* and *-4*), one mitochondrial isoform (*OsAPX5*), and three chloroplast isoforms (*OsAPX6*, *-7*, and *-8*) [10]. Wheat (*Triticum aestivum* L.) harbors 27 genes encoding APX isoforms [11,12]. Recently, functional analysis of APX genes has been carried out in tomato [13], upland cotton (*Gossypium hirsutum* L.) [14], sorghum (*Sorghum bicolor* L.) [15], maize (*Zea mays* L.) [16], *Populus trichocarpa* [17], *Brassica napus* [18], and fruit (e.g., tomatoes, sweet cherries, pears, bananas, pepper, olive, orange, grape berries, and strawberry) [19], as more plant genomes have been sequenced. These canonical APX genes are conserved across most plant species and exhibit high levels of structural and functional homology.

In higher plants, most light-regulated biological processes, including chloroplast development and photosynthesis, are complex [20]. High levels of ROS are produced in the classical water–water cycle in chloroplasts [21]. Chloroplastic canonical APX isoforms are considered the key enzyme for the reductive detoxification of H_2_O_2_ [22]. Seedling de-etiolation is the most dramatic manifestation of light-regulated plant development processes, in which *Arabidopsis* APX-R has been shown to play a direct role in germinative and post-germinative development associated with etioplast differentiation [23]. In the halophyte ice plant (*Mesembryanthemum crystallinum* L.), APX activities were detected mainly in the microsomal fraction of light-grown seedlings and the cytosolic fraction of etiolated seedlings, indicating the role of APX in light-regulated chloroplast physiological processes [24].

Recent studies have reported that APX (both canonical isoforms and APX-R/APX-L members) is also involved in responses to abiotic stresses, with distinct functional mechanisms. The *Arabidopsis* double mutant (*apx1*/*tapx*) exhibits substantial accumulation of anthocyanins under light stress, generating drastically different signals [25]. Similarly, the *apx2* mutant in rice exhibits a yellow–green leaf phenotype linked to ROS imbalance [26]. Notably, heterologous expression of *Ss.tAPX* from *Suaeda salsa* in *Arabidopsis* also enhances tolerance to high light stress [27]. In rice, the *osapx4* mutant displays a premature leaf senescence phenotype, and the dual-knockdown mutant (*sapx*/*tapx*) exhibits compromised photosystem II (PSII) activity and integrity [28]. *Arabidopsis APX4*, also known as *APX-L*, localizes to peroxisomes and plays a role in antioxidant defense by scavenging high-energy electrons originating from PSII or the oxygen-evolving complex. Previous research has shown that *atapx4* knockout mutants exhibit an increase in H_2_O_2_ peroxide accumulation [6,29]. In addition, *APX-R* (annotated as *APX6* in *Arabidopsis*) is typically targeted to chloroplasts or associated membranes, which play a role in antioxidant metabolism, contributing to oxidative protection, mainly during seed development and germination [23]. The expression level of APX is also regulated by hormones; for example, APX genes are upregulated in response to abscisic acid (ABA) treatment in maize, indicating coordinated hormonal regulation of the entire APX gene family [30].

Gene family evolution and expansion often accompany gene duplication, including tandem and segmental duplications [2,31], and this evolutionary pattern is particularly prominent in polyploid plants with complex genomic backgrounds. Oat, an important Poaceae crop, exists as diploids, tetraploids and hexaploids in nature. It is generally accepted that hexaploid oat (*A. sativa*, AACCDD) originated through hybridization events involving its ancestral species, including the diploid *A. longiglumis* (AA) and the stetraploid *A. insularis* (CCDD) [2,31]. This allopolyploidization is the core driver of homologous gene formation in gene families, which paralogous genes from intra-genomic duplication distribute across A, C, and D subgenomes with functional divergence, while orthologous genes from ancestral Poaceae retain highly conserved core sequences and functions with gramineous APX genes (wheat, rice, and other gramineous crops, etc.). Following the advent of oat genome sequencing, an increasing number of genes have been identified and functionally characterized. However, no comprehensive analysis or functional research of the APX family in *A. sativa* has been reported. Therefore, in this study, we aimed to comprehensively identify and characterize the APX gene family in the hexaploid oat (OT3098 variety) genome and characterized their physicochemical properties, gene structure, phylogenetic relationships, and duplication events. Furthermore, expression analysis of the *AsAPX* gene family in response to light was carried out. This study provides a theoretical foundation for exploring the biological functions of APX genes in oat while also offering insights for chloroplast development, photosynthetic efficiency, and overall crop performance.

## 2. Materials and Methods

### 2.1. Identification and Physicochemical Properties of APX Gene Family in A. sativa Genome

For identification of APX family members (AsAPX) in the *A. sativa* genome, the reference APX protein sequences in *Arabidopsis* were obtained from the TAIR database (https://www.arabidopsis.org/, accessed on 31 December 2024) [32]. The genome and GFF file of OT3098-v2 for cultivated *A. sativa* were downloaded from the GrainGenes database (https://graingenes.org/GG3/, accessed on 30 December 2024) [33]. *A. sativa*’s ancestors *Avena insularis* and *Avena longiglumis* were cv “BYU209_v1.1”, and cv “CN58138_v1.1” also from the GrainGenes database. TBtools (v2.091) was used to perform BLASTp alignment on the *Arabidopsis* AtAPX reference sequence [34]. A Hidden Markov Model (HMM) search with the APX gene family (PF00141) was used in a BLAST search against the *A. sativa* genome, applying an E-value threshold of 1 × 10^−5^, to identify APX genes, followed by the removal of redundant genes according to our specifications. Subsequently, TBtools was used to screen for non-redundant candidate genes, and sequence alignment validation was performed in the UniProt database (https://www.uniprot.org/, accessed on 31 December 2024) to confirm their reliability and integrity. In addition, the sequences were confirmed to contain conserved elements using the NCBI CD-Search (https://www.ncbi.nlm.nih.gov/Structure/cdd/wrpsb.cgi, accessed on 31 December 2024), ultimately identifying AsAPX gene members.

The physicochemical characteristics of the AsAPX amino acid sequences, including molecular weight, amino acid length, theoretical isoelectric point, the aliphatic index, the instability index, and the grand average of hydropathicity (GRAVY) index, were calculated using the ProtParam tool in the Expasy (https://web.expasy.org/protparam/, accessed on 3 January 2025) online portal. WoLF PSORT (https://www.genscript.com/wolf-psort.html, accessed on 3 January 2025) was used to predict AsAPX protein subcellular localization.

### 2.2. Structures and Conserved Motifs of AsAPX Gene Family

The protein-coding region (CDS) and non-coding region (UTR) of the *AsAPX* genes were extracted from the GFF3 file across the genome. Conserved motifs in AsAPX protein sequences were identified using the MEME online website (motif number = 10; motif width = 6–50 amino acids) (https://meme-suite.org/meme/tools/meme, accessed on 3 January 2025). Subsequently, TBtools (v2.091) software was employed to visualize the conserved motifs and gene structures [34,35]. The conserved motifs of AsAPX proteins were analyzed using the SeqLogo in TBtools.

### 2.3. Evolutionary and Orthologous Relationships Between AsAPX Gene Family Members

To investigate the evolutionary relationships and identify orthologous genes among APX family members across different species, phylogenetic analyses were performed. Gene sequences for members of the *Arabidopsis* APX family were downloaded from TAIR (https://www.arabidopsis.org/index.jsp, accessed on 31 December 2024), and those for *O*. *sativa* were downloaded from the rice database (https://www.ricedata.cn/, accessed on 31 December 2024). The APX sequences in other species, including *Z*. *mays*, *T. aestivum*, and *B. distachyon*, were retrieved from the Phytozome v13 database using the BLASTp tool, using *Arabidopsis* sequences as bait and a minimum threshold cutoff of e^−20^ [11,30]. The amino acid sequences of all APX target sequences were aligned using ClustalW Options in MEGA-X (10.2.2) software with default parameters (gap opening penalty = 10, gap extension penalty = 0.2), followed by the removal of the non-conserved amino acid sites that were either too short or too long in the compared protein sequences. Phylogenetic analyses were performed using the neighbor-joining (NJ) method in MEGA-X (10.2.2) software. Branch support was evaluated with 1000 bootstrap replicates; the Newick Tree file was exported and then visualized using iTOL (https://itol.embl.de/, accessed on 9 March 2026) online software. The OrthoVenn3 tool (https://orthovenn3.bioinfotoolkits.net/start/db, accessed on 9 March 2026) was utilized to infer orthologous genes between the oats and other species [36]. Based on the phylogenetic tree topology and sequence identity, homologous APX genes across species were classified.

### 2.4. Gene Duplication Events and Synteny Analysis of Orthologous Genes in AsAPX Gene Family Members

Gene duplication events, which generate paralogous genes, were analyzed using the Multicollinearity Scanning Toolkit (One Step MCScanX in TBtools (v2.091) software) with default parameters [37]. Result visualization was performed using thresholds of sequence identity ≥ 75%, E-value ≤ 1 × 10^−10^, and alignment coverage ≥ 0.75, considering that oat is an allohexaploid crop (AACCDD genome). Syntenic gene pairs identified within the oat genome were classified as segmental duplication-derived paralogous pairs, indicating a common evolutionary origin via polyploidization. To demonstrate the synteny of orthologous APX genes obtained from oat and its possible ancestors—*A. insularis* and *A. longiglumis*, dicot (*Arabidopsis*), and monocots (maize, rice, wheat, and *B. distachyon*)—a synteny analysis plot was constructed using TBtools (Dual Synteny Plotter). Additionally, the collinearity of homoeologous paralogous *AsAPX* genes (A/C/D subgenome-derived) was analyzed to explore the genomic homology and subgenome-specific retention of paralogous genes during oat allopolyploidization. Finally, the OrthoVenn3 tool (https://orthovenn3.bioinfotoolkits.net/start/db, accessed on 9 March 2026) was utilized to infer orthologous genes between the oats and other species [36].

### 2.5. Analysis of Cis-Elements in AsAPX Promoters

The promoter sequences (2000 bp upstream of the start codon) of *AsAPX*s were extracted by using TBtools software. The *cis*-element species, number, and function were analyzed using the Plant-CARE database (http://bioinformatics.psb.ugent.be/webtools/plantcare/html/, accessed on 17 March 2025). All *cis*-regulatory elements were identified, simplified, and subsequently visualized.

### 2.6. Plant Material Processing and Sample Collections

The oat variety “Dingyan No.2” was selected for this experiment. The seeds were soaked in 75% ethanol for 1 min and then in sodium hypochlorite for 5 min for disinfection, followed by thorough rinsing with distilled water. The seeds were then grown on 0.3% sucrose Murashige and Skoog media (PhytoTech (Shawnee Mission, KS, USA), M519) (pH 5.8). To establish the plant material grown in constant darkness (Dark) or light (Light), seeds were exposed to constant white light for 4 h at 24 °C to synchronize germination and then placed under darkness or white light for 7 days before sampling. The dark-grown seedlings were then transferred to white light for 24 h for de-etiolation. Etiolated seedlings illuminated for 6 h and 24 h were harvested to produce samples D7L6h and D7L24h, respectively. Leaf samples (0.5 g) were collected from 10 Petri dishes per treatment, immersed in liquid nitrogen for quick freezing, and stored in a freezer at −80 °C for later RNA extraction. Three independent biological replicates were performed.

### 2.7. AsAPX Gene Expression Patterns

Total RNA was extracted from oat pooled leaf tissues (0.5 g from 10 Petri dishes per treatment) using a FastPure Plant Total RNA Isolation Kit (Vazyme Biotech, Nanjing, China) according to the manufacturer’s instructions. First-strand cDNA was synthesized using a HiScript III 1st Strand cDNA Synthesis Kit (Vazyme Biotech, Nanjing, China) using 1 µg RNA. Quantitative reverse transcription PCR (qRT-PCR) was conducted with gene-specific primers and Taq Pro Universal SYBR qPCR Master Mix (Vazyme Biotech, Nanjing, China) on a StepOne Fast Real-Time PCR System (Applied Biosystems, Foster City, CA, USA) with three technical replicates for each of the three independent biological replicates. The PCR conditions were as follows: denaturation at 95 °C for 30 s, denaturation at 95 °C for 5 s, annealing at 57 °C for 30 s, repeated for 40 cycles, followed by 95 °C for 15 s, 60 °C for 1 min, and 95 °C for 15 s. The relative gene expression levels were calculated using the 2^−ΔΔCT^ method, with *AsGADPH* employed as the internal reference gene for normalization [38,39]. The primer sequences for qRT-PCR analysis are provided in Table S5. The 2^−∆∆Ct^ calculation was carried out according to [40].

### 2.8. Statistical Analyses

All values for qRT-PCR analysis between the control (Light) and treatment groups (Dark, D7L6h, and D7L24h) were given as means ± SD (*n* = 3), after confirming a normal distribution using the Shapiro–Wilk test. Significant differences between mean values were determined using Microsoft Excel 2019 Student’s *t*-test and are indicated with asterisks as follows: * *p* < 0.05; ** *p* < 0.01; ns, no significant difference [41,42].

## 3. Results

### 3.1. Identification and Physicochemical Analysis of the AsAPX Gene Family

Members of the *AsAPX* gene family were identified by searching the oat genome with probes derived from the eight *Arabidopsis* APX sequences and the conserved APX domain (PF00141), resulting in the identification of 27 APX members in *A. sativa*. Detailed physicochemical information about each *AsAPX* family member is summarized in Table S1. As shown in Table S1, the number of amino acids in *AsAPX* genes ranged from 112 to 510, corresponding to molecular weights of 11.83 to 55.45 kDa. The protein isoelectric point ranged from 4.84 to 12.00, with 11 proteins exhibiting isoelectric points less than 7.00, indicating that they are acidic, and the other 16 demonstrating isoelectric points greater than 7.00, classifying them as alkaline proteins. These proteins exhibited instability indices ranging from 35.52 to 96.58 and aliphatic indices ranging from 47.35 to 92.27. The average GRAVY score ranges from −0.765 (AsAPX6-1) to 0.075 (AsAPXT-2). All others were less than 0, except for AsAPXT-2, indicating that 26 APX family proteins are hydrophilic, while AsAPXT-2 is hydrophobic.

### 3.2. Chromosomal Localization of AsAPX Genes

**Figure 1:**
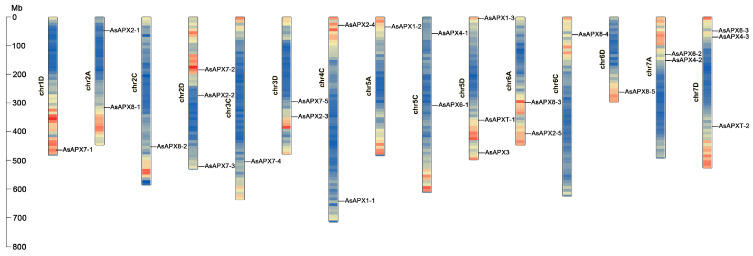
Chromosomal localization of the *AsAPX* family in *Avena sativa*. The size of each chromosome and gene position can be estimated according to the scale on the left of the figure. Chromosome colors represent gene abundance, ranging from high gene density in red to low gene density in yellow. The genome corresponding to each chromosome is sequentially labeled as chromosome 1D, 2A, 2C, 2D, 3C, 3D, 4C, 5A, 5C, 5D, 6A, 6C, 6D, 7A, and 7D.

A total of 27 AsAPX were identified and unevenly distributed across the fifteen chromosomes (Figure 1). The genes were designated as *AsAPX1-1-AsAPX1-3*, *AsAPX2-1-AsAPX2-5*, *AsAPX3*, *AsAPX4-1-AsAPX4-3*, *AsAPX6-1-AsAPX6-3*, *AsAPX7-1-AsAPX7-5*, *AsAPX8-1-AsAPX8-5*, and *AsAPXT-1-AsAPXT-2* based on their UniProt descriptions and their physical positions on the chromosomes. The distribution of these genes across the A, C, and D subgenomes was expected for an allohexaploid. Overall, *AsAPX* genes were distributed on chromosomes 1D, 2A, 2C, 2D, 3C, 3D, 4C, 5A, 5C, 5D, 6A, 6C, 6D, 7A, and 7D. Chromosomes 2D (*AsAPX2-2*, *AsAPX7-2*, and *AsAPX7-3*, located in chr2D), 5D (*AsAPX1-3*, *AsAPXT-1*, and *AsAPX3*, located in chr5D), and 7D (*AsAPX4-3*, *AsAPX6-3*, and *AsAPXT-2*, located in chr7D) harbored the most, with three *AsAPX* genes each. Chromosomes 2A, 3D, 4C, 5C, 6A, and 7A followed, harboring two *AsAPX* genes, including *AsAPX2-1* and *AsAPX8-1* located in chr2A, *AsAPX2-3* and *AsAPX7-5* located in chr3D, *AsAPX1-1* and *AsAPX2-4* located in chr4C, *AsAPX4-1* and *AsAPX6-1* located in chr5C, *AsAPX2-5* and *AsAPX8-3* located in chr6A, and *AsAPX4-2* and *AsAPX6-2* located in chr7A. Finally, six AsAPX genes (*AsAPX7-1*, *AsAPX8-2*, *AsAPX7-4*, *AsAPX1-2*, *AsAPX8-4*, and *AsAPX8-5*) were present on chromosomes 1D, 2C, 3C, 5A, 6C, and 6D, respectively. However, in this study, no corresponding *AsAPX* gene was found on Chr1A, Chr1C, Chr3A, Chr4A, Chr4D, and Chr7C of *A. sativa*. This phenomenon may be due to the frequent translocations and inversions that occurred during *A. sativa*’s polyploidization events, as previously reported [43].

### 3.3. Analysis of the AsAPX Family Gene Structure and Conserved Protein Motifs

**Figure 2:**
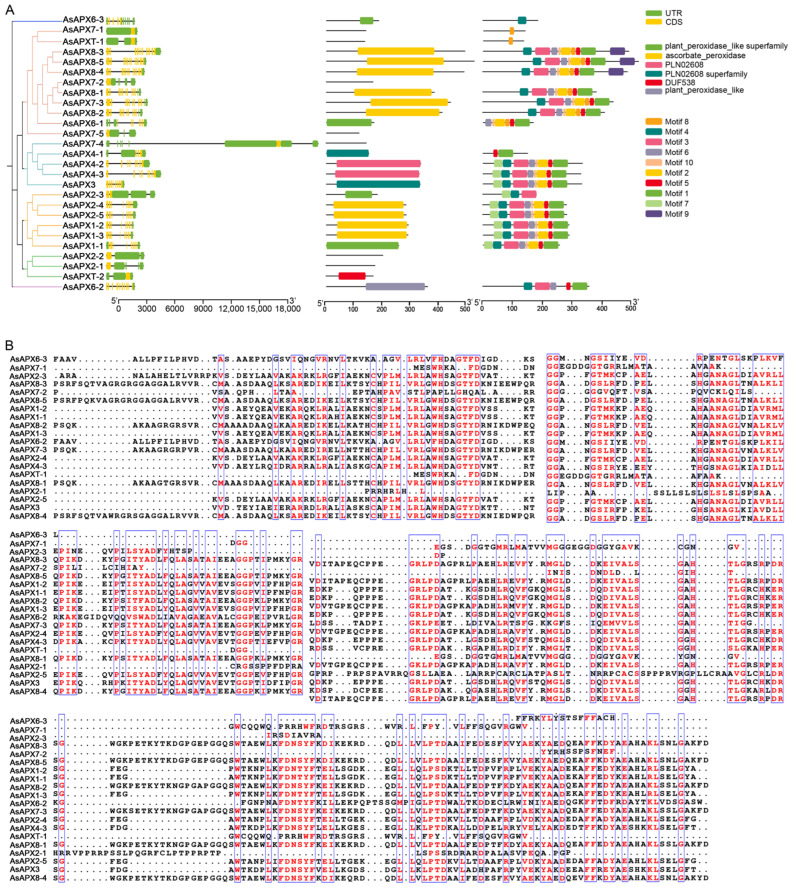
Structural analysis of *AsAPX* genes in *Avena sativa*. (**A**) Phylogenetic tree, conserved motifs, and gene structure analysis of *AsAPX* gene family. The yellow box indicates the coding region (CDS), the grey line indicates the intron, and the green box indicates the untranslated region (UTR). (**B**) Multiple sequence alignment of APX domains of the *AsAPX* gene family. Blue boxes highlight conserved regions and red text indicates highly conserved amino acid residues.

Conserved motifs and intron distribution were analyzed to improve our understanding of APX gene structural diversity (Figure 2A). The structural analysis revealed that the 5′- and 3′-UTRs varied in length among the AsAPX family members and that they comprised 1–12 exons separated by introns. *AsAPX8-3*, *AsAPX8-4*, and *AsAPX8-5* contained the most exons, while *AsAPX7-1*, *AsAPX7-2*, *AsAPXT-1*, *AsAPX7-4*, *AsAPX7-5*, *AsAPX2-1*, *AsAPX2-2*, and *AsAPXT-2* had the fewest, with only one each. A MEME analysis of the AsAPX protein sequences indicated that conserved motifs were distributed near the C-terminal region. TBtools software identified 10 conserved motifs among the 27 AsAPX proteins, and most AsAPX members shared a similar motif composition, except AsAPX7-4, AsAPX7-5, AsAPXT-2, AsAPX2-1, and AsAPX2-2.

Multiple sequence comparisons were conducted on the amino acid sequences of the AsAPX proteins, focusing on the amino acid sequences of the APX domains (Figure 2B). The analysis revealed a high level of conservation in the APX structure at the C-terminus.

### 3.4. Phylogenetic Relationships Between APX Family Genes

To investigate the evolutionary relationship of APX family genes across species, we selected various representative plant APX family genes from both diploid and polyploid plant species to construct an evolutionary tree. These included oat and its ancestors *A. insularis* and *A. longiglumis*, the eudicot model *Arabidopsis*, the monocot model rice (*O. sativa*) and maize (*Z. mays*), the model grass *Brachypodium distachyon*, and the hexaploid wheat (*T. aestivum*, AABBDD, 2n = 6x = 42), in which A/D subgenomes were conserved in both oat and wheat. *B. distachyon*, which exhibits natural ploidy diversity (predominantly diploid, 2n = 2x = 10), is widely used as a model grass due to its compact genome and conserved synteny with major cereal crops [44].

**Figure 3:**
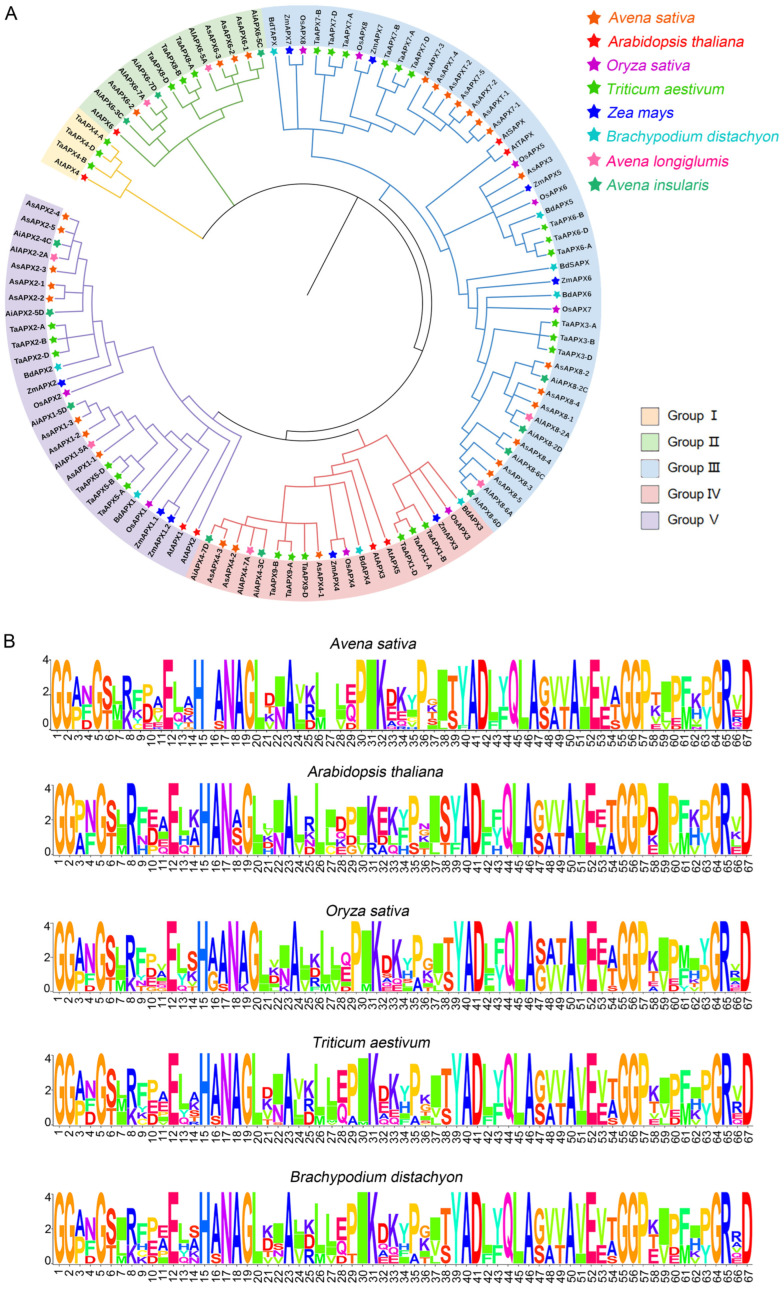
Phylogenetic analysis and conserved domain characterization of APX proteins in *Avena sativa* and other plant species. (**A**) Phylogenetic analysis of APX family genes in *Avena sativa*, *Arabidopsis thaliana*, *Oryza sativa*, *Triticum aestivum*, *Zea mays*, and *Brachypodium distachyon*, and two ancestral species, *A. longiglumis* and *A. insularis*. Different species are indicated by different colors. (**B**) Logo analysis of APX family conserved domain in *A. sativa*, *Arabidopsis*, rice, wheat, and *B. distachyon*. Sequence conservation at each position is represented by overall stack height, with relative frequency of individual residues indicated by corresponding letter height.

There are 8, 14, 8, 8, 27, 8, and 8 APX family members in *A. longiglumis*, *A. insularis*, *Arabidopsis*, *O. sativa*, *T. aestivum*, *Z. mays*, and *B. distachyon*, respectively (Figure 3A). Using MEGA-X and the NJ method, the full-length APX protein sequences from eight species were aligned, and a phylogenetic tree was constructed based on the classification of *O. sativa* and *Arabidopsis* [45]. The 27 *AsAPX* genes were divided into five groups, with each group corresponding to distinct APX isoform classes and subcellular localizations, including both classical APX categories and divergent APX-R/APX-L clades (Figure 3A). The canonical APX isoforms (Groups III–V) were predicted to be associated with chloroplasts/mitochondria, peroxisomes, and cytoplasm, respectively. Group III contains 13 *AsAPX* genes, the most among the five groups, which include *AtSAPX* and *AtTAPX*; *OsAPX5*, *OsAPX6*, *OsAPX7*, and *OsAPX8*; *AsAPX3*, *AsAPX7s*, *AsAPX8s*; and *AsAPXTs*. Group IV and V contain three and eight *AsAPX* genes, respectively. It is presumed that Group I and II APX genes are primarily associated with chloroplast development and form a distinct group of APX-like (APX-L) and APX-related (APX-R) proteins [11,29]. Among them, Group I comprises APX-L members, clustering with *Arabidopsis AtAPX4* (APX-L) and rice *OsAPX4*, which are predicted to localize to the peroxisome [6,29]. Group II contains the AsAPX6s subfamily (*AsAPX6-1*, *AsAPX6-2*, and *AsAPX6-3*), clustering with *Arabidopsis AtAPX6* and wheat *TaAPX5*, reported as APX-R members, whose function in oxidative protection is independent of canonical peroxidase activity [6,23]. Additionally, our subcellular localization prediction indicated that these AsAPX6s are targeted to the plasma membrane (chloroplastic membrane-bound form; Table S1), aligning with previous reports that APX-R proteins form dimers with chloroplast-associated APXs [23,46]. Furthermore, the phylogenetic tree revealed that all oat APX genes shared a close evolutionary relationship with their homologs in *A. insularis* and *A. longiglumis*, corroborating the ancestral origin of hexaploid oat from these two species. In terms of interspecific homology among Poaceae crops, oat and wheat APX genes were identified as the most closely evolutionarily related, followed by those in rice, consistent with their Poaceae ancestry. Interestingly, we also observed high homology between *AsAPX* and *BdAPX* genes, a phenomenon that is likely attributable to similar evolutionary selection pressures acting on these two grass species during their evolution. Overall, the *AsAPX* gene classification demonstrated clear diversification, implying distinct roles for different family members. However, genes clustered in the same group showed pronounced similarity, potentially reflecting conserved physiological functions.

Subcellular localization of the AsAPX proteins using WoLF PSORT revealed that AsAPX1 and AsAPX2 were predicted to be cytoplasmic; AsAPX7, AsAPX8, and AsAPXT were predicted to be localized to the chloroplast; and AsAPX3, AsAPX4, and AsAPX6 were predicted to be localized to the mitochondria, peroxisomes, and plasma membrane (chloroplastic membrane-bound form), respectively (Table S1). The diverse subcellular localization of APX isoenzymes suggests that they may be involved in regulating ROS in specific subcellular compartments, thereby protecting plants from oxidative damage.

To demonstrate the conservatism of sequence patterns, we performed a sequence WebLogo analysis (Figure 3B). The conserved sequence composition, amino acid types, and proportion showed high similarity among the 27 *A. sativa*, 8 *Arabidopsis*, 8 rice, 27 wheat, 8 maize, and 8 *B. distachyon* APX members (Figure 3B). Quantitative analysis of the conserved core region of the APX function domain revealed an overall sequence conservation rate of 92.7% across the six species, with key catalytic and structural residues (His, Asp, Arg) showing 100% conservation. The amino acid sequences were most similar between *A. sativa* (AACCDD) and wheat (AABBDD), with A/D subgenomes conserved in both, followed by *B. distachyon*, with a pairwise sequence identity of 91.3–94.6% (wheat) and 88.5–91.2% (*B. distachyon*) in the conserved APX domain, while the identity between oat and *Arabidopsis* was only 76.8–80.5%, further confirming the high evolutionary conservation of the APX gene family in gramineous species.

### 3.5. Collinearity Analysis of AsAPX Genes

**Figure 4:**
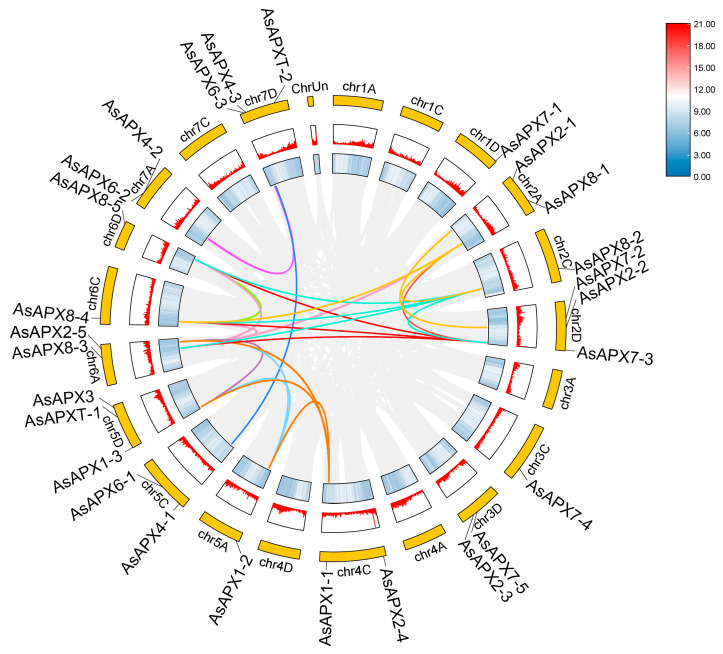
Gene duplications of APX gene family. The gray lines represent the synteny regions in the *Avena sativa* genome. The colorful lines represent syntenic *AsAPX* gene pairs.

Given that oat is an allohexaploid species (AACCDD), the identification of duplication events of APX family members in *A. sativa* was conducted using the *MCScanX* function in TBtools to explore their evolutionary relationships (Figure 4). The collinear blocks observed in the oat genome (gray lines in Figure 4) primarily reflect genomic homology (A-C, A-D, C-D) generated during polyploidization events, a key feature of *AsAPX* gene family duplication. The results showed that 16 *AsAPX* genes and 2 or more other *AsAPX* genes have fragment duplication; 24 pairs of genes derived from segmental duplication events on 12 chromosomes were visualized as colored syntenic gene pairs in collinear blocks (Figure 4, Table S2), suggesting an evolutionary relationship between these *AsAPX* gene members and potentially conserved or divergent gene functions following duplication.

**Figure 5:**
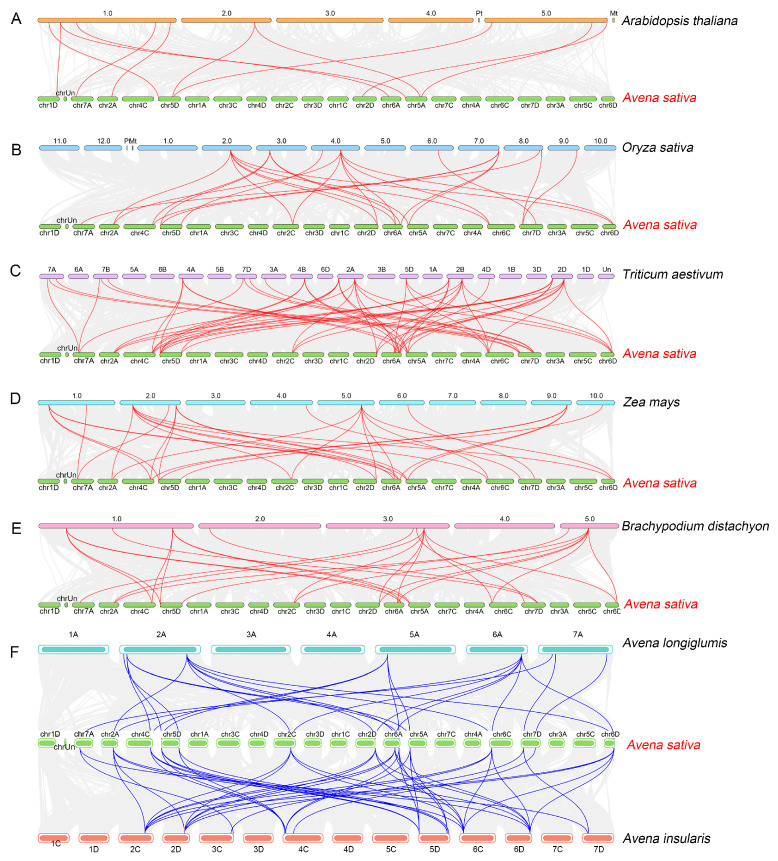
Synteny analysis of the APX genes between *Arabidopsis thaliana* and *Avena sativa* (**A**), *Oryza sativa* and *A. sativa* (**B**), *Triticum aestivum* and *A. sativa* (**C**), *Zea mays* and *A. sativa* (**D**), *Brachypodium distachyon* and *A. sativa* (**E**), and two ancestral species—*Avena longiglumis* and *Avena insularis*—and *A. sativa* (**F**). The collinear blocks within *A. sativa* and other species genomes are represented by the gray lines, while the red line and blue line highlights the colinear APX pairs.

To further deduce the evolutionary mechanism of the oat *AsAPX* gene family, we conducted a syntenic relationship analysis with oat and two ancestral species of oats (*A. insularis* and *A. longiglumis*), one eudicot (*A. thaliana*), and four monocots (rice, wheat, maize, and *B. distachyon*) (Figure 5). The results indicated that 18 and 39 *AsAPX* syntenic gene pairs were identified in *A. longiglumis* and *A. insularis*, respectively. In addition, *AsAPX* genes exhibit strong synteny with APX genes from wheat, with the highest number of homologous gene pairs observed between these species (54 pairs), which is attributed to both species being allohexaploid cereals with conserved subgenome collinearity (both AACCDD/AABBDD hexaploid genomes). There were also 11, 25, 29, and 25 homologous gene pairs between oat and *Arabidopsis*, rice, maize, and *B. distachyon*, respectively. Furthermore, to explore the homology of AsAPX with other species, we separately detected AsAPX orthologs in *A. longiglumis*, *A. insularis*, *B. distachyon*, *Arabidopsis*, wheat, rice, and maize with OrthoVenn3. Seven and ten orthologous genes were identified in *A. longiglumis* and *A. insularis*, seven in *T. aestivum*, and six each were identified in *O. sativa*, *B. distachyon*, and *Z. mays* (Table S3). The findings indicated that APX genes are highly conserved evolutionarily between oat and wheat, while oat exhibits lower synteny with dicot *Arabidopsis*, possibly due to the close genetic relationship between oat and wheat and hallmarks of polyploid evolution.

### 3.6. Cis-Acting Elements in AsAPX Genes

**Figure 6:**
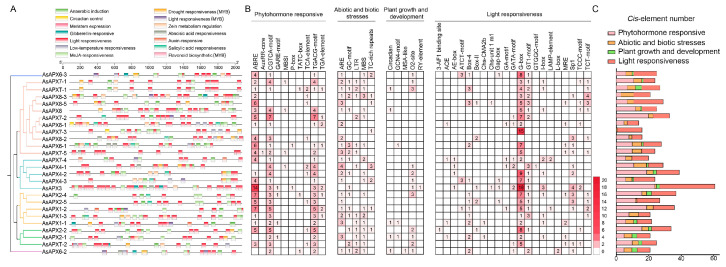
The *cis*-acting elements of the promoter region (upstream 2000 bp) of *AsAPX* genes. (**A**) Various types of *cis*-elements and their respective locations in each *AsAPX* gene. (**B**) *Cis*-element number analysis in the *AsAPX* gene family. The different intensity colors and numbers of the grid indicate the number of different promoter elements in the *AsAPX* genes. (**C**) The different colored histogram represents the sum of the *cis*-acting elements in each category.

*Cis*-acting elements play a vital role in enabling plants to adapt to environmental fluctuations and modulate growth and developmental processes [47]. Thus, to investigate the potential biological functions of *AsAPX* gene family members, a 2000 bp sequence upstream of each gene was analyzed using the PlantCARE database (Figure 6). Overall, 41 *cis*-acting elements were identified as being involved in phytohormone, abiotic, and biotic stress response, growth and development, and light responsiveness (Table S4).

Light-responsive elements include the MYB-binding site (MRE), G-box, Box-4, GT1-motif, Sp1, and the TCT-motif, which were found in approximately half of the *AsAPX* gene promoters. *AsAPX3* had the highest number (34), followed by *AsAPX7-3* (18). Abiotic and biotic stress response elements include MBS (drought), ARE (anaerobic induction), LTR (low-temperature response), defense-related TC-rich repeat, and GC-motif, which were present in approximately half of the *AsAPX* gene promoters. *AsAPX8-2* and *AsAPX6-1* had the highest number (8), whereas *AsAPX3, AsAPX7-5*, and *AsAPX2-5* had the lowest count (only one). Additionally, specific hormone response-related elements, including those for abscisic acid, auxin, gibberellin, zein, salicylic acid, and methyl jasmonate (MeJA), which play critical roles in plant growth, development, and response to environmental stress, were identified. Almost all members harbored ABRE- and MeJA-responsive (CGTCA motif) elements, suggesting that most *AsAPX* genes may be transcriptionally responsive to abscisic acid and MeJA signaling, indicating that most *AsAPX* genes are involved in plant hormone signal transduction. The total number and types of *cis*-acting elements varied among genes, reflecting the involvement of *AsAPX* genes in various biological processes. These results suggest that *AsAPX* promoters may be involved in specific transcriptional responses to light, stress adaptation, and hormonal regulation, which allows us to further infer the potential functions of *AsAPX* genes in maintaining ROS balance and regulating photomorphogenesis in oat.

### 3.7. AsAPX Gene Expression in Response to Light

**Figure 7:**
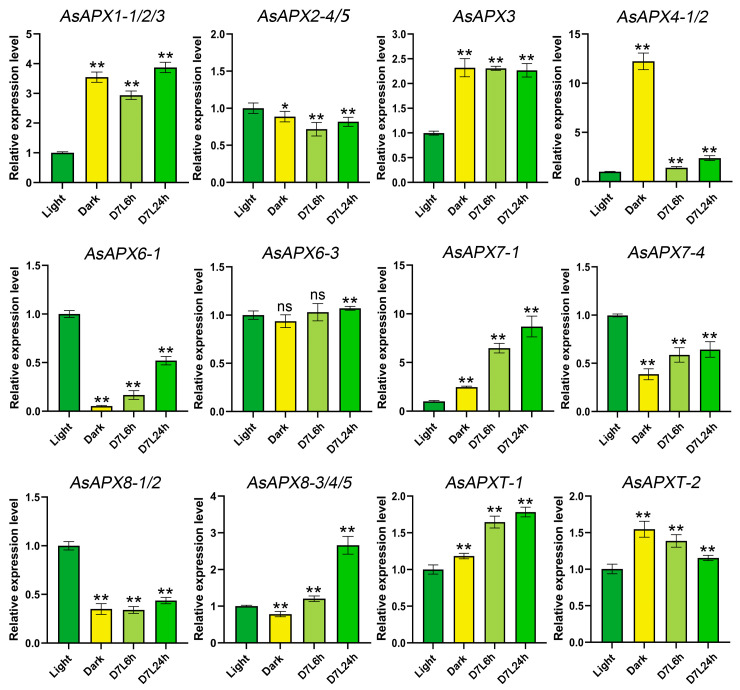
qRT-PCR analysis of light-responsive expression patterns of *AsAPX* genes. The transcript levels were measured by qRT-PCR, and expression was normalized to the *GADPH* gene. The relative expression level of each gene was calculated by using the 2^−∆∆Ct^ method. All values are given as means ± SD (*n* = 3). Significant differences between mean values determined by using Student’s *t*-test are indicated, and the significant differences between the Light and treatment groups (Dark, D7L6h and D7L24h) are indicated with asterisks as follows: * *p* < 0.05; ** *p* < 0.01; ns, no significant difference.

To explore the potential biological functions of *AsAPX* genes in response to light, seedlings were exposed to constant white light (Light) and darkness (Dark) for 7 days, followed by etiolation, and then illuminated for 6 h and 24 h (D7L6 and D7L24). The use of qRT-PCR to assess the relative transcription levels of *AsAPX*s in *A. sativa* leaves revealed different expression patterns among the genes in response to light (Figure 7). Compared with the light condition, the expression levels of most *AsAPX*s increased during the dark-to-light transition, including *AsAPX1*, *AsAPX6-1*, *AsAPX7*, *AsAPX8*, and *AsAPXT* (*p* < 0.01, Figure 7). Specifically, these genes, which exhibited similar expression levels, were predicted to be chloroplast-targeted, indicating their transcriptional responsiveness to light signals in oat leaves. By comparison, *AsAPX4* expression was high in dark conditions but low under the light treatment, suggesting its transcriptional repression by light signals. Additionally, the expression level of *AsAPX2* and *AsAPX6-3* showed little change, with 0.9- (*p* < 0.05) and 0.9-fold (ns) increases under the dark treatment, 0.7- (*p* < 0.01) and 1.0-fold (ns) increases under the D7L6 treatment, and 0.8- (*p* < 0.01) and 1.1-fold (*p* < 0.01) increases under the D7L24 treatment, respectively, demonstrating weak transcriptional responsiveness to light signals in these genes.

## 4. Discussion

As a vital global crop for both food and feed, understanding the molecular mechanisms by which *A. sativa* responds to light during development is of both scientific and practical value [1]. Light is essential to plant growth, morphology, and developmental changes [46]. Ascorbate peroxidase (APX) is one of the key enzymes associated with active oxygen removal and light-regulated plant growth and development [22]. APX genes have been characterized in various plants, including *Arabidopsis* [9], rice [48], *Populus trichocarpa* [17], and wheat [11], among others. However, most research on the APX gene family is focused on its role in removing ROS, and information on its role in the light signaling pathway is limited. This study provides a foundation for future exploration of the molecular mechanisms of APX genes and gene candidates for enhancing oat crop development through chloroplast improvement.

In this study, 27 APX gene family members in *A. sativa* were identified, each of which was found to contain a conserved ascorbic acid peroxidase domain (Figure 2). The number of *AsAPX* genes was notably higher than those reported for *Arabidopsis* and rice, but is similar to that of wheat, which reflects that the number of APX genes is related to the complexity of the plant genome [11]. Moreover, AsAPX family members exhibit a range of molecular weights, ranging from 11.83 to 55.45 kDa, with a theoretical isoelectric point (pI) of 4.84 to 12.00 (Table S1), similar to those in *A. thaliana*, rice, and wheat, indicating the evolutionary conservation of APX genes [46,49]. Analysis of the gene structures and protein motifs revealed that genes belonging to the same subtree possess similar exon–intron patterns. However, some of the genes have many exons–introns, while others lack introns; for example, *AsAPX7-4* possesses the longest structure (Figure 2A). These results are consistent with previous findings in crops such as wheat and peanut [11,50], further supporting the high evolutionary conservation of *A. sativa*.

APX enzymes can be categorized into cytoplasmic, peroxisomal, chloroplast, and mitochondrial isoforms based on their subcellular localization, while the quantity of APXs varies across different species [45]. Based on the phylogenetic tree, the 27 AsAPX members were divided into five subfamilies (I, II, III, IV, and V; Figure 3A). The phylogenetic analysis revealed that all 12 genes from subfamily III were localized to chloroplasts and/or mitochondria. Among them, *AsAPX8* genes were identified as dual-targeted to both organelles, consistent with the finding that *PtosAPX* and *AtsAPX* are dual-targeted to these two organelles [51,52]. Three genes from subfamily IV were localized to peroxisomes, while eight genes from subfamily V and three from subfamily II were found in the cytoplasm and plasma membrane, indicating that closely related genes may have had similar subcellular localization (Figure 3A and Table S1). However, the *AsAPX6s* genes, located in the plasma membrane, were separately clustered in subfamily II. This finding has been discussed in previous studies, where APX-R proteins were shown to form separate clusters and to be essential for peroxidase activity in wheat and *Arabidopsis* [11,53]. These individual genes encode membrane-bound isoforms and form a dimer with chloroplast-associated APXs but lack peroxidase activity [11,29], as demonstrated for APX-R from *Arabidopsis* (*AtAPX6*), which plays an important role in oxidative protection, primarily during seed development and germination [23,29]. These results are consistent with the analysis of chromosomal localization (Figure 1). Furthermore, *A. longiglumis* and *A. insularis* are considered ancestral to *A. sativa*, which exhibited strong evolutionary conservation of most of the genes (Figure 3A). In terms of interspecific homology among Poaceae crops, oat is evolutionarily closer to wheat, consistent with their Poaceae ancestry, which is consistent with previous studies.

The expansion of the *AsAPX* gene family is primarily attributable to polyploidization-derived segmental duplication events (Figure 4, Table S2), consistent with the known genomic architecture of hexaploid oat. The segmental duplication pairs identified in our analysis reflect the extensive collinear block retention resulting from two sequential hybridization events during oat evolution, which are direct genetic legacies of the allohexaploidization process [31]. The uneven distribution of AsAPX genes across subgenomes, with the D subgenome harboring the highest number (Figure 1), aligns with previous reports of preferential gene retention in the D subgenome following polyploidization [31]. Conversely, the absence of *AsAPX* genes on certain chromosomes likely reflects post-polyploidization chromosomal rearrangements or gene loss [2,31]. The orthologs are genes in different species that evolved from a common ancestral gene by speciation, while paralogs are genes related via duplication events within a genome [2,31]. As an important member of the Poaceae family, we further explored the evolutionary relationships of the APX gene family among *A. sativa* and rice, wheat, maize, and *B. distachyon* (Figure 5). Gene collinearity analysis among different species revealed the highest level of conservation between oat and wheat, followed by *B. distachyon*, maize, and rice, while *Arabidopsis* showed the lowest level. This may suggest a close evolutionary relationship between oat and wheat, since they are both allohexaploid. Meanwhile, AsAPX showed high homology with BdAPX, possibly due to similar selective pressures during evolution. Additionally, the total number of APX genes in *A. longiglumis* and *A. insularis* is comparable to that of *AsAPX* genes in *A. sativa*, which provides evidence for *A. longiglumis* and *A. insularis* as the putative ancestral species of *A. sativa* [2,31], suggesting that chromosomal polyploidization acts as a key driver for the origination and expansion of the *AsAPX* gene family. The paralogous genes from intra-genomic duplication distribute across A/C/D subgenomes with functional divergence, while orthologous genes from ancestral Poaceae retain highly conserved core sequences and functions with gramineous APX genes (wheat, rice, and other gramineous crops, etc.). Despite differences in ploidy, we emphasize that the APX gene family remains highly conserved in sequence (92.7% conservation across species), particularly within the Poaceae family, indicating that polyploidization expanded gene numbers without dramatically altering core functional domains [43].

It is generally accepted that promoter *cis*-elements play a crucial role in plant response to stress, light responsiveness, growth and development, and hormone responsiveness [45,54]. In this study, a 2000 bp upstream ATG sequence analysis of the AsAPX family members revealed light responsiveness-related elements such as the MYB-binding site (associated with the light responsiveness element MRE), G-box, Box-4, the GT1-motif, Sp1, and the TCT-motif, as well as stress regulatory elements, such as MBS, ARE, LTR, TC-ric, and the GC-motif (Figure 6). Notably, the number of light-responsive elements in their promoters (e.g., *AsAPX7*s, *AsAPX8*s) had a certain relationship with light-inducible expression of qRT-PCR data, while those lacking such elements (*AsAPX4*s) did not (Figure 6 and Figure 7). This concordance provides functional validation of the *cis*-element predictions. Additionally, nearly all *AsAPX* genes contain ABRE and MeJA-responsive *cis*-elements, implying the potential regulatory responsiveness of the APX gene family to abscisic acid and methyl jasmonate-mediated signaling pathways. Each *AsAPX* gene contains at least one phytohormone- and one plant growth and development-related *cis*-element. Notably, *AsAPX7*, *AsAPX8*, and *AsAPXT* were all strongly induced in leaves during the transition from dark to light (Figure 7), indicating their pronounced transcriptional responsiveness to light signals.

Light serves as a signal for plant growth and development throughout their entire life cycle, during which seedling de-etiolation is a major process involving the production of ROS [3]. It is known that APX is critically involved in regulating physiological processes, detoxifying photoproduced H_2_O_2_, and responding to diverse abiotic stresses [22,55]. In this study, qRT-PCR was used to appraise the light-responsive expression patterns of *AsAPX* genes during plant development under different light conditions (Figure 7). Previous research has shown that the expression of t-APX increases during de-etiolation and the dark-to-light transition in spring wheat seedlings, enabling them to mitigate oxidative stress during this process [24,56]. Consistent with this, the transcriptional levels of *AsAPX7s*, *AsAPX8s*, and *AsAPXTs* showed a continuous increase for 24 h under dark-to-light conditions, although the increase in *AsAPX7-4* and *AsAPX8-1/2* expression was lower than under the light treatment, which indicates that the chloroplast-targeted *AsAPX* genes are transcribed in seedlings grown under the dark-to-light transition; in contrast, *AsAPX4-1/2* expression peaked under the dark conditions before declining, suggesting that these genes may function primarily transcriptionally repressed by light and may exhibit dark-specific transcriptional activity in peroxisomal-associated antioxidant pathways. Furthermore, the expression of *AsAPX1s*, *AsAPX2s*, *AsAPX6-3*, and *AsAPX3* was detected in both light-grown and dark-to-light-grown seedlings, indicating that these cytosolic and microsomal APX isoforms maintain mild light-induced transcriptional levels.

## 5. Conclusions

This study identified and characterized the 27 APX gene family in hexaploid *A. sativa*, providing fundamental information for further functional studies of *AsAPX* genes in oat growth, and photomorphogenesis under light/dark treatment. Gene structures and conserved motifs were highly conserved among orthologous members. The phylogenetic analysis and subcellular localization prediction enabled AsAPX proteins to be classified into five categories. APX and APX-R were shown to be conserved while exhibiting functional and expression profiling divergence. Most members of the AsAPX (oat), AiAPX (*A. insularis*), and AlAPX (*A. longiglumis*) gene families display strong evolutionary conservation. The formation and expansion of the *AsAPX* gene family in oat are likely driven primarily by gene duplication events and gene fragment recombination. In addition, qRT-PCR analyses indicated the roles of the *AsAPX* genes in response to light. This study provides an enhanced understanding of the evolution and functions of the APX gene family in *A. sativa*, presenting a framework for further detailed analysis and exploration. It paves the way for the development of stress-resistant transgenic crop plants and additional studies of this gene family.

## Data Availability

Data are contained within this article and the Supplementary Materials.

## Supplementary Materials

The following supporting information can be downloaded at: https://www.mdpi.com/article/10.3390/life16030494/s1, Table S1: Data for 27 *AsAPX* genes identified in *Avena sativa* genome; Table S2: Detailed information about syntenic APX gene pairs; Table S3. The inferred AsAPX orthologs in *Avena longiglumis* and *Avena insularis* and other species; Table S4: List of *cis*-acting elements of *AsAPX* gene family members; Table S5: Sequences of primers used in the study.

## Abbreviations

The following abbreviations are used in this manuscript:

APX	Ascorbic peroxidase
ROS	Reactive oxygen species
PSII	Photosystem II
ABA	Abscisic acid
MRE	MeJA, methyl jasmonate
CDS	Coding region
UTR	Non-coding region
NJ	Neighbor-joining
